# A novel online calculator based on clinical features and hematological parameters to predict total skin clearance in patients with moderate to severe psoriasis

**DOI:** 10.1186/s12967-023-04847-4

**Published:** 2024-01-31

**Authors:** Yuxiong Jiang, Dawei Huang, Qianyu Chen, Yingyuan Yu, Yifan Hu, Yu Wang, Rongfen Chen, Lingling Yao, Xiaoyuan Zhong, Luyang Kong, Qian Yu, Jiajing Lu, Ying Li, Yuling Shi

**Affiliations:** 1 Department of Dermatology, Shanghai Skin Disease Hospital, Tongji University School of Medicine, No. 1278 Bao de Road, Shanghai, 200443 China; 2 Institute of Psoriasis, Tongji University School of Medicine, Shanghai, China; 3 Department of Dermatology, Shanghai Tenth People’s Hospital, Tongji University School of Medicine, Shanghai, China

**Keywords:** Psoriasis, Total skin clearance, Psoriasis area and severity index, Nomogram, Predictive modeling

## Abstract

**Background:**

Treatment responses to biologic agents vary between patients with moderate to severe psoriasis; while some patients achieve total skin clearance (TSC), a proportion of patients may only experience partial improvement.

**Objective:**

This study was designed to identify potential predictors for achieving TSC in psoriasis patients treated with IL-17 inhibitors. It also aimed to develop an easy-to-use calculator incorporating these factors by the nomogram to predict TSC response.

**Methods:**

A total of 381 patients with psoriasis receiving ixekizumab were included in the development cohort and 229 psoriasis patients who initiated secukinumab treatment were included in the validation cohort. The study endpoint was achieving TSC after 12 weeks of IL-17 inhibitors treatment, defined as the 100% improvement in Psoriasis Area and Severity Index (PASI 100). Multivariate Cox regression analyses and LASSO analysis were performed to identify clinical predictors and blood predictors respectively.

**Results:**

The following parameters were identified as predictive factors associated with TSC: previous biologic treatment, joint involvement, genital area affected, early response (PASI 60 at week 4), neutrophil counts and uric acid levels. The nomogram model incorporating these factors achieved good discrimination in the development cohort (AUC, 0.721; 95% CI 0.670–0.773) and validation cohort (AUC, 0.715; 95% CI 0.665–0.760). The calibration curves exhibited a satisfactory fit, indicating the accuracy of the model. Furthermore, the decision curve analysis confirmed the clinical utility of the nomogram, highlighting its favorable value for practical application. Web-based online calculator has been developed to enhance the efficiency of clinical applications.

**Conclusions:**

This study developed a practical and clinically applicable nomogram model for the prediction of TSC in patients with moderate to severe psoriasis. The nomogram model demonstrated robust predictive performance and exhibited significant clinical utility.

*Trial registration* A multi-center clinical study of systemic treatment strategies for psoriasis in Chinese population;ChiCTR2000036186; Registered 31 August 2020; https://www.chictr.org.cn/showproj.html?proj=58256.

**Supplementary Information:**

The online version contains supplementary material available at 10.1186/s12967-023-04847-4.

## Introduction

Psoriasis is a chronic inflammatory skin disease that affects approximately 2–3% of the world’s population. It not only causes physical discomfort and pain but also has a significant impact on health-related quality of life (HRQoL), leading to psychological distress and social isolation [1, 2]. Despite the availability of various treatment options, including topical corticosteroids, phototherapy, and systemic medications, many patients with plaque psoriasis do not achieve satisfactory control of their symptoms [3]. In recent years, biologic agents have revolutionized the treatment of plaque psoriasis by targeting specific components of the immune system involved in the pathogenesis of the disease [4].

In psoriasis clinical trials for biologic agents, a 90% improvement in psoriasis area and severity index (PASI 90) is widely accepted as a technical treatment goal [5]. However, in psoriasis patients who respond to treatment but without total skin clearance (TSC), the residual disease may continue to have negative impacts on HRQoL and increase the risk of comorbidities such as psoriatic arthritis (PsA). Multiple studies have indicated that patients with psoriasis who achieved PASI 100 experienced significantly greater improvements in HRQOL and reduced pruritus symptoms than those with almost skin clearance (PASI 90–100) [6–8]. Therefore, achieving TSC represents a clinically meaningful treatment goal in daily practice, especially from the patient’s perspective.

Interleukin (IL)-17 A inhibitors, such as ixekizumab and secukinumab have emerged as an effective treatment option for plaque psoriasis. Clinical trials have demonstrated the remarkable efficacy of IL-17 inhibitors in the treatment of plaque psoriasis, with a significant proportion of patients achieving PASI100 after 12 weeks of treatment [9]. However, not all patients respond equally to treatment, and predicting individual treatment outcomes remains a challenge [10]. In order to optimize the effectiveness of anti-IL17 therapies, it is crucial for clinicians to identify predictive factors that can assist in determining the patients who are most likely to derive substantial benefits.

Identifying patients who are more likely to respond to IL-17 inhibitors has significant clinical implications. It enables personalized treatment decisions, ensuring optimal therapy selection and potentially reducing treatment failures, healthcare costs, and improving patient outcomes [11, 12]. In this study, we will develop a nomogram based on the logistic regression model to provide a visual representation of the predictive factors and their corresponding probabilities of achieving TSC. The findings of this study hold significant implications for tailoring the management of psoriasis on an individualized basis, thereby potentially advancing the development of enhanced therapeutic approaches.

## Methods

### Study design and setting

This real-world prospective, multicenter observational cohort study included psoriasis patients in the dermatological centers and outpatient clinics of 26 general hospitals in China between September 2020 to May 2022. Inclusion criteria were listed as follows: (1) patients aged 18 years or older; (2) patients diagnosed with moderate to severe psoriasis, confirmed by a dermatologist; (3) patients who provide written informed consent to participate in the study; (4) patients who have completed 12 weeks of biologic treatment. Exclusion criteria: (1) patients who are currently participating in another clinical trial involving an investigational drug or therapy; (2) patients who have administered IL-17 inhibitors in the past; (3) patients with severe infections or immunodeficiency disorders.

A development cohort including 381 psoriasis patients treated with ixekizumab was used for identifying the independent predictive factors for TSC response and to develop a predictive model. Patients received ixekizumab 160 mg at week 0.80 mg at weeks 2 and 4, then 80 mg every 4 weeks up to and including week 12. A total of 229 patients with psoriasis receiving secukinumab were included in the development cohort. Patients received secukinumab 300 mg once a week from 0 to 4 weeks then 300 mg every 4 weeks up to and including week 12. The disease’s severity and treatment response were evaluated by body surface area (BSA), PASI, and dermatology quality of life index (DLQI) at baseline and after 4 and 12 weeks. Ethical approval for the study was approved by the Clinical Research Ethics Committee of the Shanghai Skin Disease Hospital (approval #2020-36), in compliance with the Declaration of Helsinki. All patients in the study provided informed consent for the review of their clinical data.

### Data collection

To minimize bias, a standardized data collection protocol was implemented. The following demographic and clinical data were obtained: Age (years), Sex, Duration of psoriasis (year), Age at onset of psoriasis (year), Bodyweight (kg), Baseline PASI score, Baseline BSA score, Baseline DLQI score, History of comorbidities (hypertension, hyperlipidemia, diabetes mellitus, obesity), Prior treatment history (systemic non-biologic treatments, phototherapy, biologic agents) and Special areas involvement (joints, nails, scalp, palmoplantar area and genital area). All laboratory tests were performed at a central laboratory using standardized laboratory procedures.

### Variables analyze and model development

Appropriate statistical methods were employed to minimize bias in the data analysis. Data collection involved the gathering and analysis of clinical and hematological parameters. Continuous variables were summarized using median and interquartile range (IQR) and compared using Wilcoxon rank-sum tests. Categorical variables were presented as counts and percentages, and their comparison was conducted using either Chi-square tests or Fisher’s exact tests, depending on the suitability of each test for the specific variable. Receiver operating characteristic (ROC) analysis was performed to assess the predictive power of early PASI response at week 4 for determining TSC at week 12. The value of PASI percentage improvement with the highest predictive value was determined by calculating the Youden Index (YI) at each percentage of PASI improvement (a), which is represented by the equation YI (a) = sensitivity (a) + specificity (a)– 1. Spearman r was calculated and *P* < 0.05 suggested a highly relevant association. Multivariable logistic regression and LASSO logistic regression were applied respectively for clinical variables and hematological parameters to identify meaningful candidate variables. Based on multivariate logistic regression analysis, the selected variables were developed into a prediction model that was presented as a nomogram.

### Model assessment

The discriminative ability of the model was evaluated using the area under the ROC curve (AUC). Calibration was assessed by conducting a Hosmer-Lemeshow goodness-of-fit test after dividing the sample into quintiles. This test was employed to determine the extent to which the model accurately fits the observed data, with a p-value greater than 0.05 indicating no indication of poor fit. The calibration curves, aligning with the 45-degree line, demonstrated an exceptional calibration model wherein the predicted probabilities closely matched the actual outcomes. In order to assess the clinical efficacy of the nomogram model, decision curve analysis (DCA) was conducted by evaluating the net benefit within a specified range of threshold probabilities. The statistical analyses were conducted using R software (version 3.6.1). ROC curves were generated using the ‘pROC’ package, while nomograms and calibration curves were created using the ‘rms’ package. DCA was generated using the ‘rmda’ package.

## Results

### The heterogeneous responses to IL-17 inhibitors

A total of 381 psoriasis patients provided baseline blood samples and had 12-week follow-up data were included in our analysis. The evaluation of the treatment response includes physician reported outcomes (PASI) and patient-reported outcomes (DLQI). After 12 weeks treatment, among 381 ixekizumab-treated patients, 43.0% of patients (*n* = 164) achieved a PASI 100, while about 6.0% of patients (*n* = 23) did not achieve PASI 75. In terms of DLQI improvement, 58.5% of patients (*n* = 232) achieved a DLQI 0–1, corresponding to having no effect on QoL; while 17.3% of patients (*n* = 65) scored a DLQI score greater than 5, corresponding to having moderate to severe effect on QoL (Additional file 1: Table S1).

### Identify patient’s characteristics and early response as clinical predictors for TSC

The baseline demographic and disease factors of the TSC and non-TSC groups are shown in Table 1. Compared with controls who failed to achieve TSC, patients who achieved TSC were more often females (35.4 vs. 22.1%, *p* = 0.004), had lower bodyweight (67.75 vs. 72 kg, *p* = 0.014), had less previous use of biologic treatments (4.9 vs. 12.4%, *p* = 0.011), had fewer joints affected (28.7 vs. 44.7%, *p* = 0.001), had less nails affected (25.0% vs. 38.7%, *p* = 0.005), had fewer genital area affected (6.7 vs. 15.2%, *p* = 0.010).

**Table 1:** Comparison of baseline patients and disease characteristics between TSC groups and non-TSC groups

Characteristics	Total (*n* = 381)	TSC (*n* = 164)	Non-TSC (*n* = 217)	p-value
Age (years), median (IQR)	37 (29, 49)	35 (28.75, 47)	38 (29, 50)	0.175
Female Sex, n (%)	106 (27.8%)	58 (35.4%)	48 (22.1%)	0.004
Age at onset of psoriasis (year), median (IQR)	27 (20, 37)	26 (18.75, 34)	27 (21, 39)	0.064
Duration of psoriasis (year), median (IQR)	7 (2, 14)	8 (3, 15)	7 (2, 14)	0.266
Bodyweight (kg), median (IQR)	70 (61, 80)	67.75 (58, 78.25)	72 (64, 81)	0.014
Baseline PASI score, median (IQR)	15 (10.3, 21.6)	14.5 (10.15, 19.15)	15.28 (10.4, 25.2)	0.079
Baseline BSA score, median (IQR)	20 (10, 35.6)	19.8 (10.38, 34.48)	21 (10, 40)	0.244
Baseline DLQI score, median (IQR)	12 (7, 18)	10 (6.75, 16.25)	12 (7, 18)	0.165
Comorbidities				
Hypertension, n (%)	45 (11.8%)	17 (10.4%)	28 (12.9%)	0.447
Hyperlipidemia, n (%)	77 (20.2%)	27 (16.5%)	50 (23.0%)	0.113
Diabetes mellitus, n (%)	22 (5.8%)	9 (5.5%)	13 (6.0%)	0.835
Obesity, n (%)	66 (17.3%)	23 (14.0%)	43 (19.8%)	0.139
Prior psoriasis treatments				
Previous systemic non-biologic treatments, n(%)	215 (56.4%)	92 (56.1%)	123 (56.7%)	0.909
Previous phototherapy, n (%)	71 (18.6%)	38 (23.2%)	33 (15.2%)	0.048
Previous biologic treatments, n (%)	35 (9.2%)	8 (4.9%)	27 (12.4%)	0.011
Psoriasis involvement				
Joints affected, n (%)	144 (37.8%)	47 (28.7%)	97 (44.7%)	0.001
Nails affected, n (%)	125 (32.8%)	41 (25.0%)	84 (38.7%)	0.005
Scalp affected, n (%)	322 (84.5%)	137 (83.5%)	185 (85.3%)	0.646
Palmoplantar area affected, n (%)	102 (26.8%)	39 (23.8%)	63 (29.0%)	0.252
Genital area affected, n (%)	44 (11.5%)	11 (6.7%)	33 (15.2%)	0.010

*TSC* total skin clearance, *IQR* interquartile range, *PASI* Psoriasis Area and Severity Index, *BSA* body surface area, *DLQI* Dermatology Quality of Life Index

**Fig. 1:**
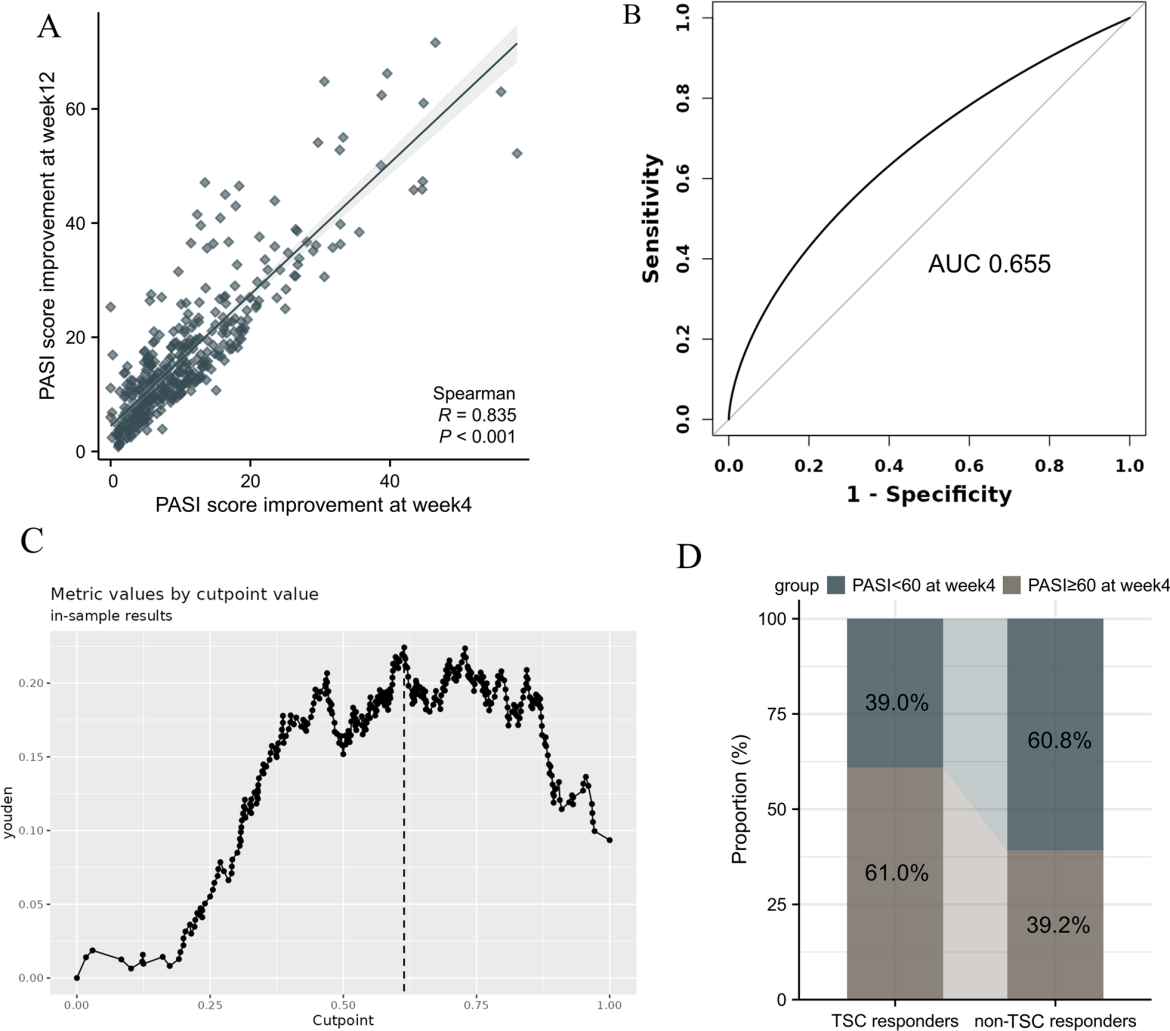
Predictive value of week 4 PASI percentage improvement for predicting week 12 TSC responders. **A** The Spearman correlation between week 4 PASI score improvement and week 12 PASI score improvement. **B** The receiver operating characteristic (ROC) curves for PASI percentage improvement at week 4 predict TSC response at week 12. **C** Youden Index for percent change from baseline PASI at week 4. PASI60 response at week 4 had the highest Youden Index and was determined as the optimum threshold for predicting TSC responders. **D** Differences in the proportion of patients reaching PASI60 at week 4 between TSC and non-TSC responders. *PASI* Psoriasis Area and Severity Index, *TSC* total skin clearance

We further explored the association of partial clearance at week 4 with achieving TSC at the end of therapy. Figure 1A shows that there was a significant positive correlation between week 4 PASI score improvement and week 12 PASI score improvement (*r* = 0.835, *p* < 0.001). The best cutoff value of the week 4 PASI percentage improvement for predicting TSC was further calculated by ROC Curve analysis and Youden’s index (Fig. 1B). As shown in Fig. 1C, a 60% improvement in PASI score from baseline (PASI 60) at week 4 achieved the highest Youden index and thus was the optimum threshold for predicting a TSC. Among TSC responders, 61.0% of patients achieved PASI 60 at week 4, which was significantly higher than 39.2% found in non-TSC responders (Fig. 1D).

**Fig. 2:**
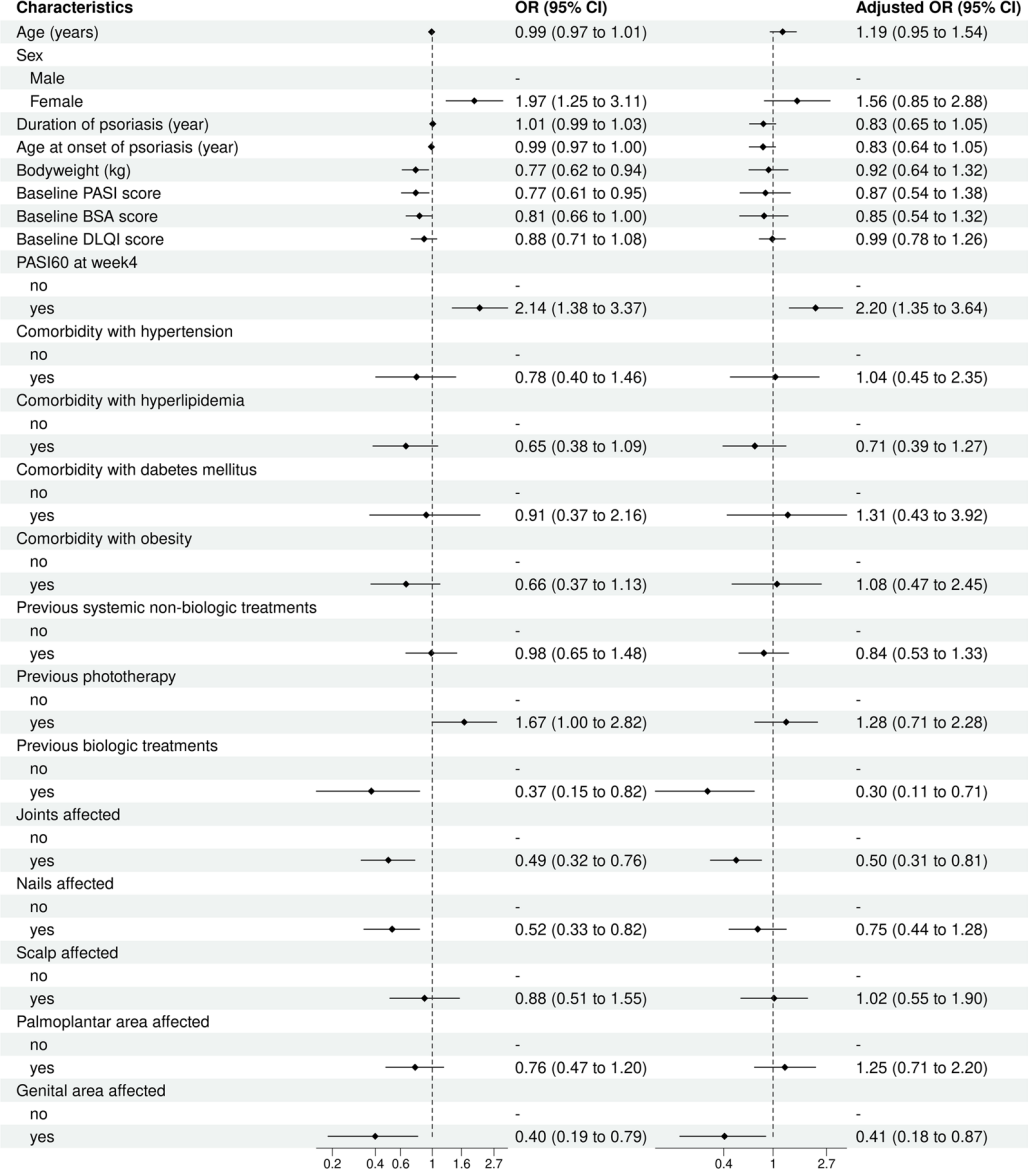
Multivariate analyses of clinical predictors associated with TSC response in psoriasis. *PASI* Psoriasis Area and Severity Index, *BSA* body surface area, *DLQI* Dermatology Quality of Life Index, *TSC* total skin clearance

Afterward, multivariate logistic regression analyses were performed to identify clinical predictors associated with TSC. As a result, previous biologic use (odds ratio (OR) = 0.30, 95% confidence interval (CI) = 0.11–0.71), joints affected (OR 0.50; 95% CI 0.31–0.81), genital area affected (OR 0.41; 95% CI 0.18–0.87) and achieving PASI 60 at week 4 (OR 2.20; 95% CI 1.35–3.64) could be an independent predictor of TSC (Fig. 2).

### Identify pre-treatment hematological parameters as blood predictors for TSC

**Fig. 3:**
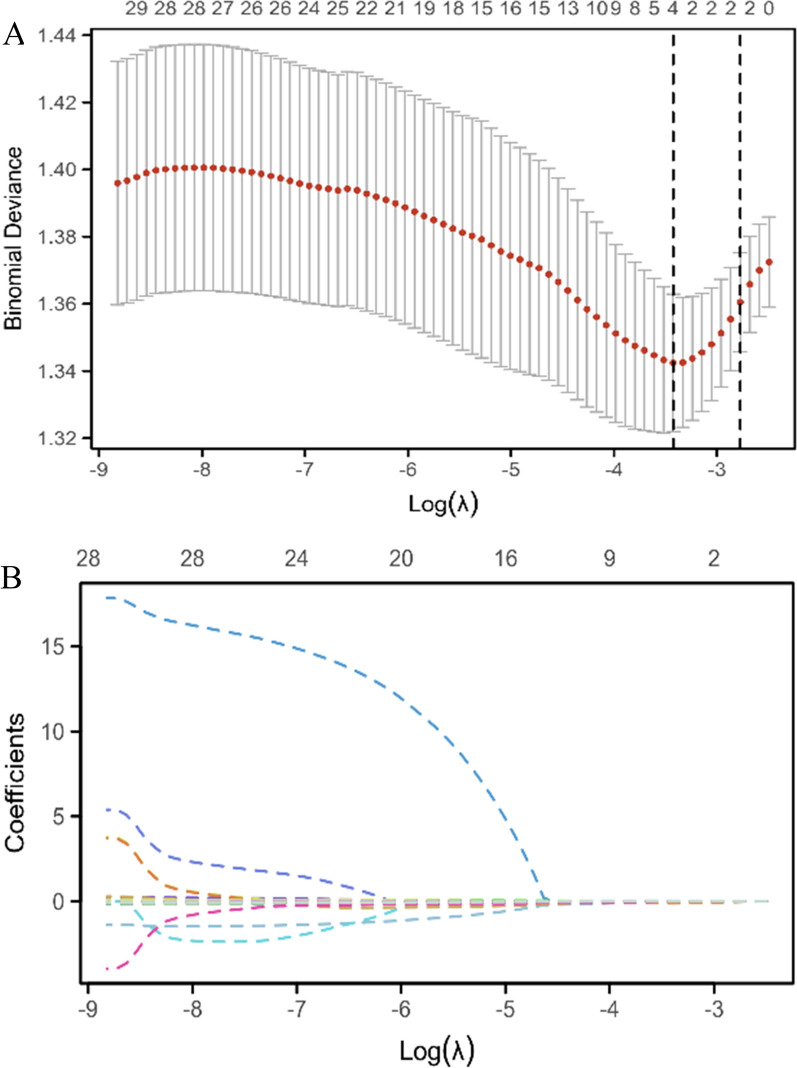
Hematological predictors are selected by the least absolute shrinkage and selection operator (LASSO) algorithm. **A** The LASSO estimates the profile of predictive variables. The penalization parameter λ was selected using 10-fold cross-validation based on the minimum criteria. The left vertical line indicates the optimal lambda location and the right vertical line indicates 1 standard error of optimal lambda. **B** Plot of the LASSO coefficient profiles

As shown in Table 2, the pre-treatment laboratory blood parameters of psoriasis patients are summarized. The WBC counts (*p* = 0.012), neutrophil counts (*p* = 0.004), CRE concentration (*p* = 0.040) and uric acid level (*p* = 0.019) were significantly different between TSC responders and non-TSC responders (Table 2). We found strong significant associations among some hematological parameters (*r* = – 0.45–0.91) (Additional file 1: Fig S1). Therefore, LASSO analysis was adopted to reduce the effects of collinearity among hematological parameters, and screen for best predictors from candidate indexes. The model that exhibited the highest level of regularity and simplicity, with a cross-validated error within one standard error of the minimum, incorporated two variables, as indicated by the right vertical dashed line (Fig. 3). Among candidate blood indexes, neutrophil counts and uric acid levels were selected as potent predictors of TSC response by LASSO analysis.

**Table 2:** Comparison of pre-treatment routine blood tests and blood biochemical tests between TSC groups and non-TSC groups

Characteristics	Total	Non-TSC	TSC	p-value
	(*n* = 381)	(*n* = 217)	(*n* = 164)	
Routine blood tests				
RBC, ×10^6^/µL	4.92 (4.39, 5.54)	4.95 (4.39, 5.51)	4.90 (4.45, 5.35)	0.330
MCH, pg	30.80 (29.40, 31.70)	30.80 (29.40, 31.60)	30.75 (29.30, 31.70)	0.973
MCHC, g/dL	337 (328, 343)	337 (329, 344)	336 (327.75, 342)	0.416
MCV, µm^3^	91.00 (88.00, 93.80)	90.90 (88.00, 93.40)	91.15 (87.98, 94.00)	0.602
Hematocrit, %	44.00 (39.60, 46.90)	43.61 (38.10, 46.70)	44.25 (40.70, 47.20)	0.091
Hemoglobin level, g/dL	150 (136, 160.6)	150 (139, 161)	149 (136, 160)	0.440
WBC, /µL	6.85 (5.77, 8.14)	7.14 (6.00, 8.37)	6.54 (5.50, 7.86)	0.012
Neutrophils, ×10^9^/L	4.09 (3.30, 5.11)	4.35 (3.54, 5.31)	3.86 (3.12, 4.87)	0.004
Lymphocytes, ×10^9^/L	1.97 (1.59, 2.34)	1.93 (1.57, 2.41)	1.98 (1.62, 2.28)	0.701
Monocytes, ×10^9^/L	0.44 (0.35, 0.59)	0.47 (0.36, 0.61)	0.43 (0.33, 0.55)	0.065
Basophil, ×10^9^/L	0.03 (0.02, 0.05)	0.03 (0.02, 0.05)	0.03 (0.01, 0.04)	0.209
Eosinophils, ×10^9^/L	0.14 (0.08, 0.22)	0.14 (0.09, 0.22)	0.15 (0.08, 0.23)	0.930
Platelet, ×10^3^/µL	248 (213, 287)	252 (215, 292)	243 (212.75, 283)	0.264
Blood biochemical tests				
ALT level, U/L	23.00 (15.00, 35.50)	23.00 (15.00, 35.50)	22.67 (14.00, 35.32)	0.332
AST level, U/L	20.83 (17.60, 25.45)	21.00 (17.72, 26.40)	20.51 (17.37, 23.49)	0.122
ALP level, U/L	78.45 (70.00, 85.00)	78.22 (70.00, 86.05)	79.36 (68.00, 84.08)	0.718
γ-GGT level, U/L	29.18 (19.00, 44.75)	31.1 (19.43, 47.00)	27.70 (18.00, 40.02)	0.105
Albumin level, g/dL	46.00 (43.80, 47.80)	45.88 (43.60, 47.90)	46.2 (43.88, 47.69)	0.423
Globulin level, g/dL	28.40 (26.84, 30.17)	28.54 (27.01, 30.51)	28.065 (26.65, 29.60)	0.060
TBIL, mg/d	11.50 (9.10, 14.10)	11.90 (9.30, 14.50)	11.32 (9.06, 13.63)	0.101
DBIL, mg/dL	3.40 (2.70, 4.20)	3.56 (2.83, 4.60)	3.345 (2.60, 4.04)	0.054
IBIL, mg/d	8.07 (6.60, 9.60)	8.07 (6.30, 10.00)	8.07 (6.90, 9.18)	0.430
FPG level, mmol/l	5.28 (5.04, 5.52)	5.28 (5.04, 5.56)	5.27 (5.04, 5.52)	0.619
TC level, mmol/L	4.75 (4.39, 5.21)	4.75 (4.36, 5.31)	4.74 (4.41, 5.11)	0.636
TG level, mmol/L	1.63 (1.24, 2.25)	1.73 (1.25, 2.25)	1.54 (1.23, 2.21)	0.365
LDL-C, mmol/L	3.00 (2.63, 3.37)	3.00 (2.58, 3.50)	2.99 (2.70, 3.24)	0.474
HDL-C, mmol/L	1.15 (1.08, 1.22)	1.15 (1.06, 1.22)	1.15 (1.09, 1.22)	0.462
UA level, mg/dL	388.29 (291.08, 485.50)	401.78 (296.76, 506.80)	377.04 (291.27, 462.81)	0.019
CRE concentration, mg/dL	72.63 (64.00, 81.71)	73.42 (65.41, 82.80)	71.38 (62.98, 78.36)	0.040

*RBC* red blood cell, *MCH* mean corpuscular hemoglobin, *MCHC* mean corpuscular hemoglobin concentration, *MCV* mean corpuscular volume, *WBC* white blood cell, *ALT* alanine transaminase, *AST* aspartate transaminase, *ALP* alkaline phosphatase, *γ-GGT* γ-glutamyltransferase, *TBIL* total bilirubin, *DBIL* direct bilirubin, *IBIL* indirect bilirubin, *FPG* fasting plasma glucose, *TC* total cholesterol, *TG* triglyceride, *LDL-C* low-density lipoprotein-cholesterol, *HDL-C* high-density lipoprotein-cholesterol, *UA* uric acid, *CRE* creatinine

### Nomogram development for TSC prediction in psoriasis patients

**Fig. 4:**
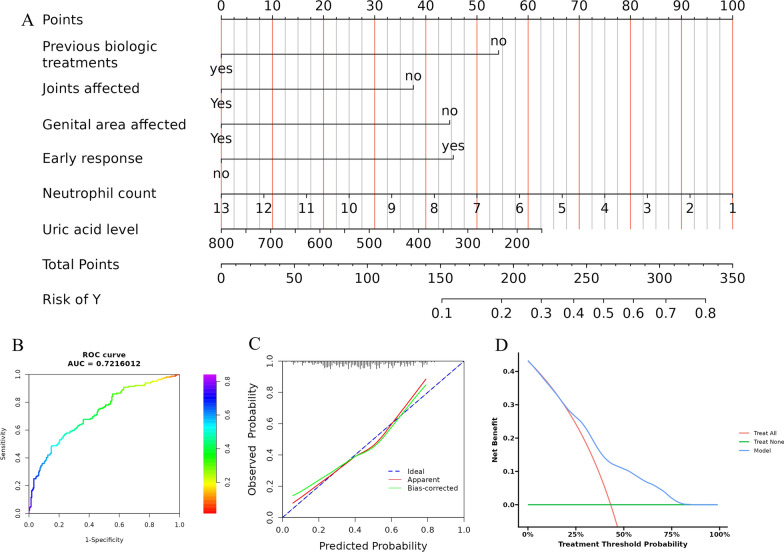
The display and evaluation of the nomogram model for predicting TSC response.** A** A nomogram predicting the probability of TSC response. **B** Receiver-operating characteristic curves for the TSC nomogram. **C** The calibration curves for the TSC nomogram. The 45◦ dashed line is a reference line that shows perfect prediction. The red dotted line is the performance of the nomogram, while the green solid line corrects for any bias in the nomogram. **D** Decision-curve analysis for the TSC nomogram. The y-axis represents the net benefit and the x-axis shows the threshold probability of response. The ‘Treat All’ line refers to the hypothesis that all patients were treated with ixekizumab and the ‘Teart None’ line to the assumption that no patient was treated with ixekizumab. *TSC* total skin clearance

The final prediction model was built up by combining with all selected clinical and hematological predictors, including previous biologic treatment, joint involvement, genital area affected, early response (PASI 60 at week 4), neutrophil counts and uric acid levels, and integrating them into a multivariable model. To utilize the nomogram model, a vertical line was drawn upwards to the points axis for each predictor, the points from each predictor were aggregated, and a vertical line was subsequently drawn downwards from the total points axis to ascertain the probability of TSC response (Fig. 4A). The nomogram model demonstrated its reliability in predicting the probability of TSC response, as evidenced by an AUC of 0.721 (95% CI 0.670–0.773) (Fig. 4B). The P-values obtained from the Hosmer- Lemeshow test were greater than 0.05 (*P* = 0.804), indicating that the multivariable model exhibited a good fit to the data. The calibration curves further confirmed a significant concordance between the predicted TSC probability and the observed TSC rate (Fig. 4C). We used DCA to analyze the clinical usefulness. The results of decision curve analysis (DCA) indicated that utilizing the nomogram for predicting TSC response, particularly when the threshold probability ranged from 20 to 80%, yielded greater net benefit compared to the “treat all” or “treat none” strategies (Fig. 4D).

### Validation of the prediction model

**Fig. 5:**
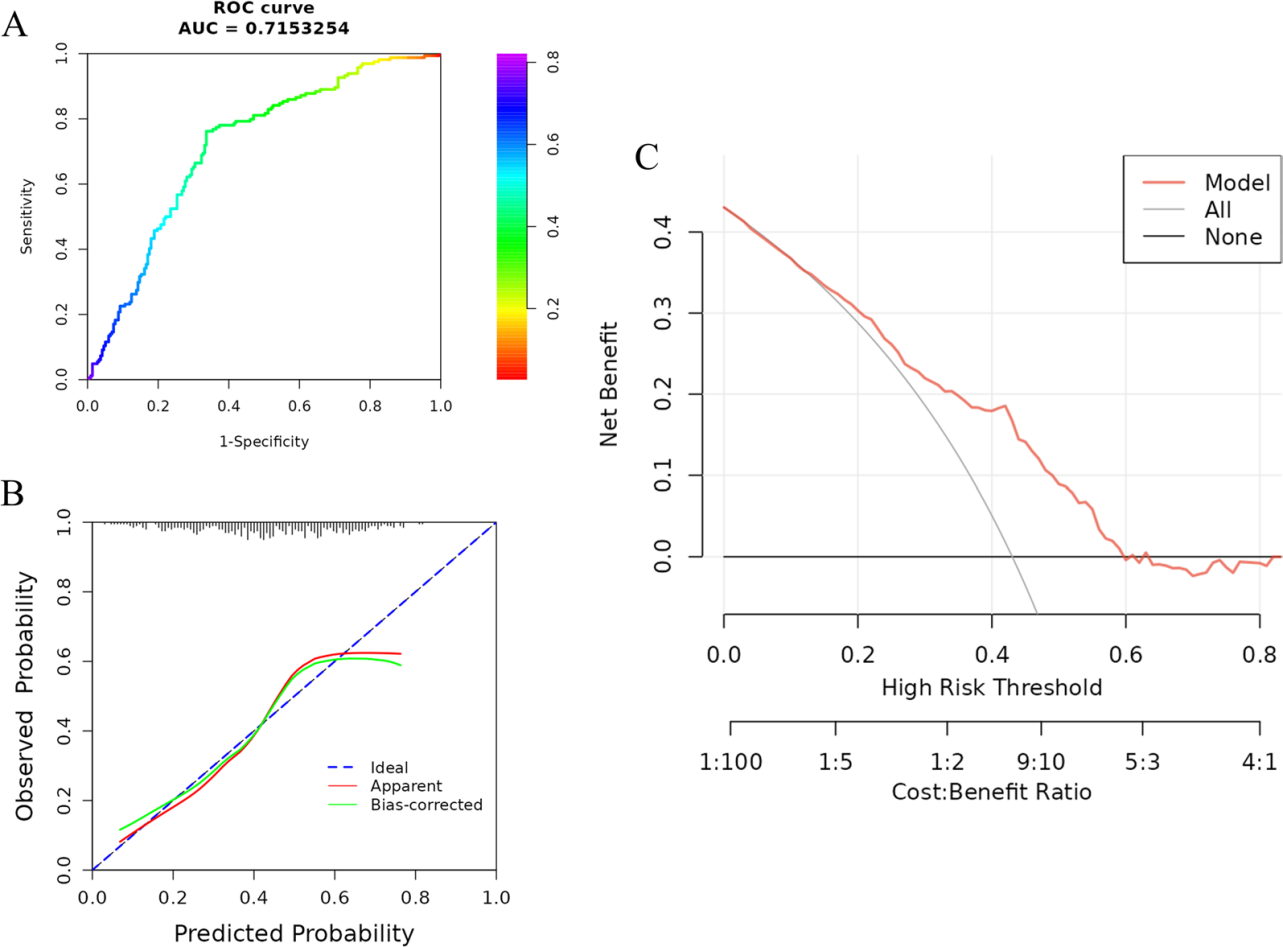
Performance evaluation of the model in the validation cohort. **A** Receiver operating characteristic to assess the ability of nomogram to predict TSC response. **B** Calibration curves of the nomogram model. **C** Decision curve analysis of the nomogram model

To validate the predictive model, we included a validation cohort of 229 psoriasis patients treated with secukinumab. There were no significant differences in baseline demographic and disease characteristics between the development cohort and the validation cohort (Additional file 1: Table S2). The ROC analysis revealed that the nomogram model exhibited a good discrimination ability in the validation cohort, with an AUC of 0.715 (95% CI 0.665–0.760) (Fig. 5A). The calibration curve demonstrated favorable agreement between the actual observations and the nomogram predictions in the validation cohort (Fig. 5B). Additionally, the Hosmer-Lemeshow test did not provide evidence of poor model fit (*P* = 0.34). Furthermore, the DCA demonstrated the superior performance of the predictive model over a wide range of threshold probabilities (Fig. 5C).

### Development of a web-based online calculator

**Fig. 6:**
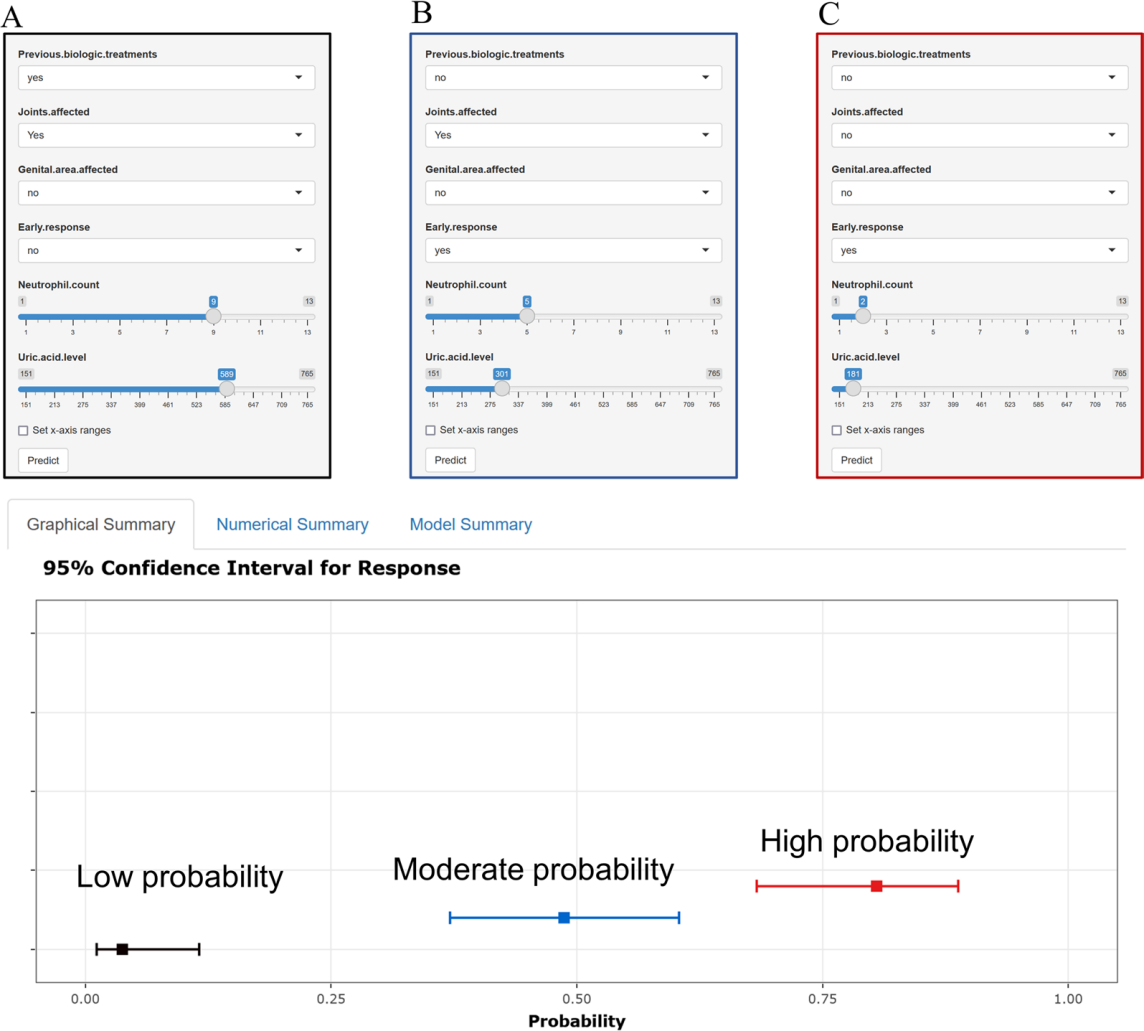
Web-based online calculator for the probabilities of TSC response. **A**–**C** Examples of low, moderate, and high probabilities of TSC response. *TSC* total skin clearance

To make it easier for clinicians to use this model, we set up a free web-based online calculator based on the shinyapp.io platform. When users simply input the requested information on predictors, the probability of TSC can be derived. This easy-to-use prediction calculator may assist patients and physicians in decision-making regarding anti-IL-17 therapy. Examples of the low, moderate, and high probabilities of TSC response are displayed in Fig. 6. This online calculator is available at https://nomopvqbgwa.shinyapps.io/dynnomapp.

## Discussion

Currently, the therapeutic objective for individuals with psoriasis has progressed from achieving remission to attaining total skin clearance (TSC), which represents a more ambitious goal [6, 13]. As a ‘treat-to-target’ strategy, TSC improves the quality of life and pruritus symptoms, prolongs drug survival, and decreases the risks of complications such as psoriatic arthritis [14, 15]. In this multicenter real-world study, fewer than half of all patients could achieve a TSC response after 12 weeks of IL-17 inhibitors treatment. At present, no single index can be used to predict TSC response [16]. Therefore, it is necessary to combine routine clinical data and laboratory parameters to predict the treatment response.

Previous studies have suggested that certain biomarkers may be associated with treatment response in psoriasis. For instance, female sex has been found to be associated with a better response to systemic therapy in psoriasis compared with males, it can be partly explained by weight, adherence to treatment and different lifestyle behavior [17]. In the present data, we also observed that TSC responders had a higher proportion of females compared with non-TSC responders. Unfortunately, it was not influential enough to enter the final model through multivariable logistic regression analysis. Thus, physicians should not make decisions according to patient’s gender. Consistent with past studies, we found that experience of prior biologic treatment could affect response to ixekizumab [18, 19]. This phenomenon can potentially be attributed to the prolonged inhibition of a specific cytokine, leading to the induction of other pro-inflammatory cytokines with overlapping functions. Manifestations of plaque psoriasis can occur in special areas, making it difficult to treat [20, 21]. Our findings agree with others that involvement with joints and genital area affected were associated with reduced odds of achieving TSC response.

In randomized controlled trials (RCT) for IL-17 A inhibitors in psoriasis, the correlation between early improvements in disease activity and improved long-term clinical outcomes has been observed. This is demonstrated by pooled data from phase 3 studies of secukinumab, which indicate that an early onset of response, defined as a PASI 50 at week 4, is associated with sustained efficacy at week 16 [22]. Similarly, in another post-hoc analysis of phase II study for ixekizumab, early PASI40 response at week 4 was predictive of PASI 75 response at week 12 [23]. However, there is limited real-world evidence available in this particular area. Results of the current analysis confirm previous findings showing that early response could serve as a reliable indicator for later response. Furthermore, our results complement and extend previous studies that a 60% improvement in PASI from baseline to week 4 was the optimum value for predicting PASI 100 response at week 12.

The routine blood test, a widely available and fundamental examination, has long been advocated as an indispensable adjunct for disease assessment. The combination of clinical characteristics and laboratory parameters may make the prediction model more accurate and effective [24, 25]. Among multiple routine blood indexes, baseline neutrophil counts and uric acid levels were found to be the best biomarkers for predicting TSC in our analysis. TNF-α accelerates the infiltration of neutrophils from the peripheral blood into the skin with dendritic cell activation [26]. The severe psoriasis group exhibited elevated neutrophil activity in the bloodstream compared to the moderate psoriasis group, and this activation was inhibited by biologic therapy in the psoriasis patients [27]. Recently, multiple studies have shown that the neutrophil-based index could serve as predictive biomarkers of treatment response to biologic agents in patients with psoriasis [28, 29]. Additionally, psoriasis patients commonly present with high levels of uric acid, which have been shown to facilitate inflammatory pathways through the release of pro-inflammatory chemokines [30]. Several studies have shown that serum uric acid concentration in psoriasis patients is positively associated with disease severity and extent of skin involvement [31, 32]. During 52 weeks of treatment with secukinumab, uric acid levels decreased in psoriasis patients [33]. A study by Pan et al. demonstrated that pre-treatment uric acid was effective in predicting the responses to biologic agents in patients with Crohn’s disease [34]. The mechanisms underlying the association between neutrophil counts and uric acid levels with treatment response to biologic agents need to be further investigated.

Collectively, we have identified six factors that exhibit predictive capabilities for the TSC response, specifically, previous biologic treatment, joint involvement, genital area affected, early response, neutrophil counts, and uric acid levels. Our findings signify a significant advancement in the stratification of psoriasis patients during the initial stages of IL-17 inhibitors treatment, thereby facilitating a personalized approach to the prescription of IL-17 inhibitors. Furthermore, the development of a web-based online calculator enhances the accessibility and efficiency of the nomogram in clinical practice. Clinicians can easily input patient characteristics into the calculator to obtain individualized predictions of TSC response. This tool can aid treatment decision-making, facilitate patient counseling, and optimize the allocation of healthcare resources.

Our study has several strengths. Firstly, the involvement of six dermatology centers throughout China guarantees the external validity of the findings. The second is to take into consideration predictive factors from three different aspects: patient and disease characteristics (previous biologic treatment, joint involvement and genital area affected), early treatment response (achieving PASI60 at week 4) and serological biomarker (neutrophil counts and uric acid levels), which is a promising approach to improve the predictive accuracy of the combined model. Third, we included only routine clinical and laboratory data in our study, thereby obviating the need for additional physical examinations or genetic profiling of patients. Several limitations should be considered when interpreting the results of this study. The study is based on data collected in the daily routine and some data were missing. Unlike in RCT, selection bias and potential confounders are inevitable, and many patients did not strictly follow the visit schedule in the study. Second, the study focused on IL-17 inhibitors, and the generalizability of the nomogram to other biologic agents or systemic therapies requires further investigation. Further, only short intervention course (12 weeks) was examined.

## Conclusion

This study established a novel model for predicting TSC response to anti-IL17 therapies in psoriasis patients. This model included six factors significantly associated with TSC response: previous biologic treatment, joint involvement, the genital area affected, early response, neutrophil counts, and uric acid levels. We believe that this novel nomogram can inform the optimization of individual treatment options for psoriasis patients and contribute to the improvement of their clinical outcomes.

### Supplementary Information

**Additional file 1: Figure S1.** The Spearman correlation analysis between pre-treatment hematological variables. RBC: red blood cell; MCH: mean corpuscular hemoglobin; MCHC: mean corpuscular hemoglobin concentration; MCV: mean corpuscular volume; WBC: white blood cell; ALT: alanine transaminase; AST: aspartate transaminase; ALP: alkaline phosphatase;γ-GGT: γ-glutamyltransferase; TBIL: total bilirubin; DBIL: direct bilirubin; IBIL: indirect bilirubin; FPG: fasting plasma glucose; TC: total cholesterol; TG: triglyceride; LDL-C: low-density lipoprotein-cholesterol; HDL-C: high-density lipoprotein-cholesterol; UA: uric acid; CRE: creatinine. **Table S1.** Psoriasis patients treated with ixekizumab showed different levels of skin clearance and quality of life improvement. **Table S2.** Clinical baseline characteristics in the development and validation cohorts.

## Data Availability

All data generated or analyzed during this study are included in this article. Further enquiries can be directed to the corresponding author.
