# Supplementary material for: A novel online calculator based on clinical features and hematological parameters to predict total skin clearance in patients with moderate to severe psoriasis
Source: J Transl Med. 2024 Jan 31;22:121. doi: 10.1186/s12967-023-04847-4 (PMC10829231; doi:10.1186/s12967-023-04847-4)
Supplement: Supplementary file 1 — Additional file 1: Figure S1. The Spearman correlation analysis between pre-treatment hematological variables. RBC: red blood cell; MCH: mean corpuscular hemoglobin; MCHC: mean corpuscular hemoglobin concentration; MCV: mean corpuscular volume; WBC: white blood cell; ALT: alanine transaminase; AST: aspartate transaminase; ALP: alkaline phosphatase;γ-GGT: γ-glutamyltransferase; TBIL: total bilirubin; DBIL: direct bilirubin; IBIL: indirect bilirubin; FPG: fasting plasma glucose; TC: total cholesterol; TG: triglyceride; LDL-C: low-density lipoprotein-cholesterol; HDL-C: high-density lipoprotein-cholesterol; UA: uric acid; CRE: creatinine. Table S1. Psoriasis patients treated with ixekizumab showed different levels of skin clearance and quality of life improvement. Table S2. Clinical baseline characteristics in the development and validation cohorts. [file 12967_2023_4847_MOESM1_ESM.docx]

**Additional file**

**Figure S1. The Spearman correlation analysis between pre-treatment hematological variables.** RBC: red blood cell; MCH: mean corpuscular hemoglobin; MCHC: mean corpuscular hemoglobin concentration; MCV: mean corpuscular volume; WBC: white blood cell; ALT: alanine transaminase; AST: aspartate transaminase; ALP: alkaline phosphatase;γ-GGT: γ-glutamyltransferase; TBIL: total bilirubin; DBIL: direct bilirubin; IBIL: indirect bilirubin; FPG: fasting plasma glucose; TC: total cholesterol; TG: triglyceride; LDL-C: low-density lipoprotein-cholesterol; HDL-C: high-density lipoprotein-cholesterol; UA: uric acid; CRE: creatinine.

**
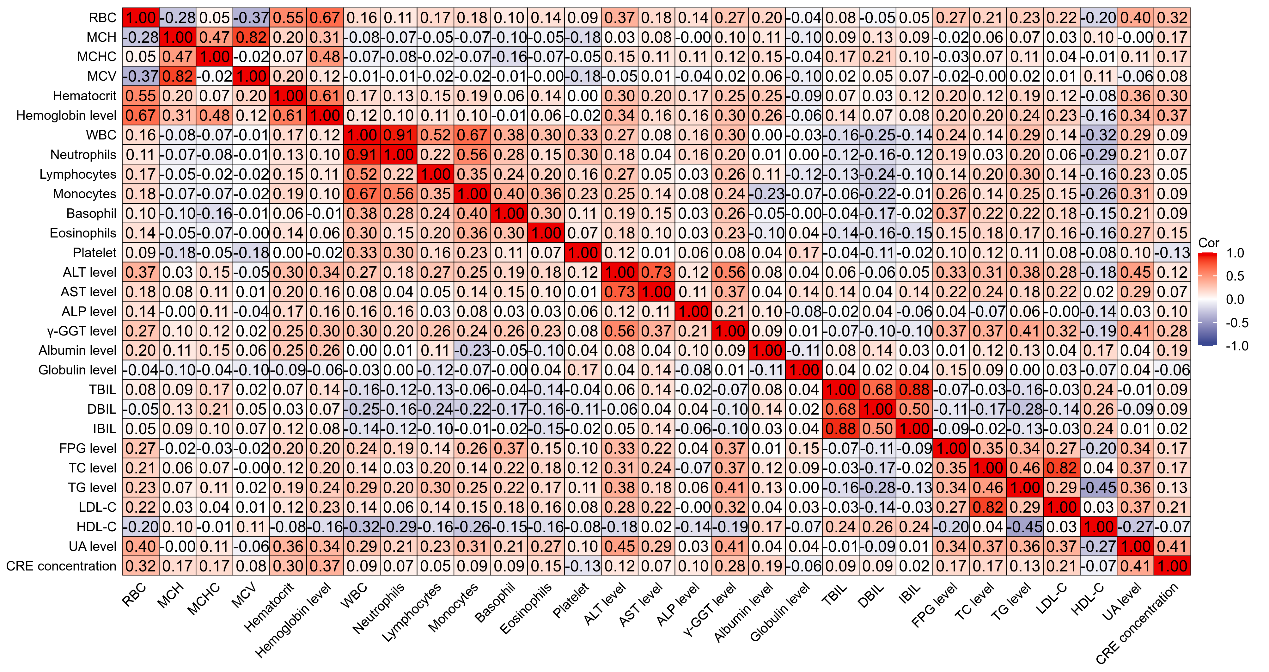
**

Table S1. Psoriasis patients treated with ixekizumab showed different levels of skin clearance and quality of life improvement.

|  | **Week 4** | **Week 12** |
| --- | --- | --- |
| **PASI (n, %)** |  |  |
| PASI 100 (clear) | 11 (2.9%) | 164 (43.0%) |
| PASI 90<100 (almost clear) | 23 (6.0%) | 128 (33.6%) |
| PASI 75<90 (partial clear) | 75 (19.7%) | 66 (17.3%) |
| PASI <75 (inadequate response) | 272 (71.4%) | 23 (6.0%) |
| **DLQI (n, %)** |  |  |
| 0-1 (No effect) | 81 (21.3%) | 223 (58.5%) |
| 2-5 (Small effect) | 106 (27.8%) | 90 (23.6%) |
| 6-10 (Moderate effect) | 127 (33.3%) | 44 (11.5%) |
| 11-20 (Very large effect) | 55 (14.4%) | 21 (5.5%) |
| 21-30 (Extremely large effect) | 12 (3.1%) | 3 (0.8%) |

PASI: Psoriasis Area and Severity Index; DLQI: Dermatology Quality of Life Index.

Table S2. Clinical baseline characteristics in the development and validation cohorts.

| characteristics | Development cohort  (n=381) | Validation cohorts (n=229) | p-value |
| --- | --- | --- | --- |
| Age (years), median (IQR) | 37 (29, 49) | 39 (29, 50) | 0.083 |
| Female Sex, n (%) | 106 (27.8%) | 54 (24%) | 0.230 |
| Age at onset of psoriasis (year), median (IQR) | 27 (20, 37) | 27 (20, 39) | 0.352 |
| Duration of psoriasis (year), median (IQR) | 7 (2, 14) | 8 (4, 14) | 0.127 |
| Bodyweight (kg), median (IQR) | 70 (61, 80) | 72 (63, 81) | 0.072 |
| Baseline PASI score, median (IQR) | 15 (10.3, 21.6) | 15 (10, 24) | 0.270 |
| Baseline BSA score, median (IQR) | 20 (10, 35.6) | 19 (9, 34) | 0.482 |
| Baseline DLQI score, median (IQR) | 12 (7, 18) | 11 (7, 17) | 0.360 |
| **Comorbidities** |  |  |  |
| Hypertension, n (%) | 45 (11.8%) | 29 (12.7%) | 0.338 |
| Hyperlipidemia, n (%) | 77 (20.2%) | 53 (23.1%) | 0.080 |
| Diabetes mellitus, n (%) | 22 (5.8%) | 14 (6.1%) | 0.727 |
| Obesity, n (%) | 66 (17.3%) | 45 (20%) | 0.141 |
| **Prior psoriasis treatments** |  |  |  |
| Previous systemic non-biologic treatments, n (%) | 215 (56.4%) | 133 (58%) | 0.426 |
| Previous phototherapy, n (%) | 71 (18.6%) | 34 (15%) | 0.053 |
| Previous biologic treatments, n (%) | 35 (9.2%) | 25 (11%) | 0.121 |
| **Psoriasis involvement** |  |  |  |
| Joints affected, n (%) | 144 (37.8%) | 92 (40.2%) | 0.192 |
| Nails affected, n (%) | 125 (32.8%) | 79 (34.5%) | 0.074 |
| Scalp affected, n (%) | 322 (84.5%) | 193 (84.3%) | 0.876 |
| Palmoplantar area affected, n (%) | 102 (26.8%) | 65 (28.4%) | 0.383 |
| Genital area affected, n (%) | 44 (11.5%) | 28 (12.2%) | 0.229 |

TSC: total skin clearance; IQR: interquartile range; PASI: Psoriasis Area and Severity Index; BSA: body surface area; DLQI: Dermatology Quality of Life Index.
